# Better by Wrapping, Yet Worse from Heat

**Published:** 1896-04

**Authors:** H. C. Morrow


### BETTER BY WRAPPING, YET WORSE BY HEAT.

EDITOR HOMŒOPATHIC PHYSICIAN:

I have a patient with the peculiar symptom, pains in neck and head better by wrapping it up in a shawl, but worse by heat of fire. I find this symptom on page 194, Vol. IX, HOMŒOPATHIC PHYSICIAN, under Rhus-tox., but cannot find it any other place. Will you please ask the profession through THE HOMŒOPATHIC PHYSICIAN if any other remedies have this peculiar modality?

Yours fraternally,
H. C. MORROW.

